# Real-world evidence of the effectiveness and utilization of subcutaneous C1INH long-term prophylaxis in patients with HAE in Spain and Germany

**DOI:** 10.3389/fimmu.2025.1576235

**Published:** 2025-05-14

**Authors:** Marcus Maurer, Stefan Cimbollek, Maebh Kelly, Kyle Rodney, John Elliott, Eduardo LoboGuerrero, Markus Magerl

**Affiliations:** ^1^ Angioedema Center of Reference and Excellence (ACARE), Institute of Allergology, Charité – Universitätsmedizin Berlin, Berlin, Germany; ^2^ Fraunhofer Institute for Translational Medicine and Pharmacology (ITMP), Immunology and Allergology, Berlin, Germany; ^3^ Allergy Department, Angioedema Reference Center (CSUR), Hospital Universitario Virgen del Rocío, Sevilla, Spain; ^4^ CSL Behring, Haywards Heath, United Kingdom; ^5^ Adivo Associates, San Francisco, CA, United States

**Keywords:** hereditary angioedema, prophylaxis, C1INH, real-world evidence, long-term, subcutaneous, switch

## Abstract

Hereditary angioedema (HAE) types 1/2 are rare genetic disorders leading to C1 inhibitor (C1INH) deficiency/dysfunction. Guidelines recommend long-term prophylaxis (LTP) to prevent HAE attacks. Subcutaneous (SC) C1INH replacement therapy is approved for LTP in patients with HAE (age indication varies between countries). There is little real-world data on the outcomes of patients who switch to C1INH SC in Europe, particularly those who switch from C1INH IV. This retrospective patient chart analysis captured real-world evidence of the effectiveness of C1INH SC LTP in patients with HAE in Germany (n=69) and Spain (n=37). The primary endpoint was change in annualized attack rate (AAR) in patients who used C1INH IV LTP during a 6-month baseline period and switched to C1INH SC LTP for ≥6 months. Switching to C1INH SC LTP from C1INH IV LTP was associated with a 73.2% reduction in AAR (n=48; P<0.001) compared to baseline. Emergency Room (ER) visits and rescue medication use were also significantly reduced after switching to C1INH SC LTP from C1INH IV LTP. A similar reduction in AAR (68.9%), ER visits (49.8%), and rescue medication use (61.9%) was observed in the overall population (n=105), regardless of treatment at baseline. Similar changes from baseline were seen in patients from Germany and Spain.

## 1 Introduction

Hereditary angioedema (HAE) is a rare genetic condition, characterized by recurrent, debilitating and potentially life-threatening cutaneous or submucosal edema (1). Pathogenic variants in the *SERPING1* gene are the predominant cause of HAE, resulting in a deficiency in functional C1 inhibitor (C1INH), a key regulator of the kallikrein-kinin cascade, responsible for bradykinin production. In patients with HAE-C1INH, bradykinin overproduction leads to increased vascular permeability and subsequent angioedema (1). There are now a number of treatment options for the management of HAE-C1INH, including on-demand (OD) treatment to reduce the duration and severity of symptoms, and long-term prophylaxis (LTP), which aims to prevent the occurrence of attacks (2). Available treatment mechanisms of action include: i) replenishing the absent/deficient C1INH with human plasma-derived or recombinant C1INH, ii) blocking the action of bradykinin by targeting its B2 receptor, or iii) inhibiting kallikrein – another mediator responsible for bradykinin release (2).

The World Allergy Organization/European Academy of Allergy and Clinical Immunology (WAO/EAACI) guideline was updated in 2021 to state that the ultimate goals of treatment should be to achieve complete control of the disease and to normalize patients’ lives – goals which can currently only be achieved by LTP. As such, the guideline recommends that all patients are evaluated for LTP at every visit, considering disease activity, burden, control and patient preference. It is also recommended that patients who use LTP are assessed regularly, as these factors can vary over time (3).

LTP with intravenous (IV) C1INH (Cinryze^®^; Takeda) is a well-tolerated treatment that reduced the frequency of HAE attacks in a crossover study from 12.73 attacks in the 12-week placebo period to 6.26 attacks in the 12-week IV C1INH LTP period (P<0.001) (4). Mean duration and severity of attacks were also significantly reduced (4). Despite efficacy of IV C1INH in trials, maintaining venous access can be challenging for some patients, and is associated with adverse effects such as infection, occlusion and thrombosis, particularly if a central venous port is used (5, 6). Based on the phase 3 COMPACT study (7), in 2017, human plasma-derived C1INH for subcutaneous (SC) injection (Berinert^®^ 2000/3000 [Haegarda^®^, US]; CSL Behring) was approved for self-administration for the prevention of recurrent attacks in patients with HAE by the FDA and several European countries (indication varies between countries from ‘≥6 years old’ to ‘adolescents and adults’) (8). In the placebo-controlled crossover trial, LTP with C1INH SC was well tolerated, and displayed significant efficacy versus placebo; median monthly attack rate was reduced by 95%, and 90% of patients receiving 60 IU/kg C1INH SC experienced a ≥50% reduction in the number of attacks versus placebo (7). C1INH SC reduced the rate of HAE attacks by 3.5 attacks per month (P<0.001) and reduced the need for rescue medication from 3.89 uses per month during the placebo period to 0.32 during the 60 IU/kg C1INH SC period (P<0.001) (7). In addition to reducing attack burden, patients using C1INH SC in the COMPACT trial and its open label extension (OLE) reported improved health-related quality of life (QoL) scores following treatment (9, 10), with HAE-QoL questionnaire scores for patients taking C1INH SC being close to the maximum (best) possible score (9). Interestingly, significant QoL improvements were observed despite around 60% of patients having received prior LTP at the start of the OLE study (9).

A subgroup analysis (n=12) of the COMPACT study, on patients that received IV C1INH at baseline, found that switching from C1INH IV LTP to SC C1INH LTP resulted in a reduction in HAE attack rate of 53.7% (11). This was supported by an indirect comparison of LTP C1INH IV and LTP C1INH SC treatment effects in the CHANGE and COMPACT studies, respectively, which reported a significantly greater mean percent reduction in monthly attack rate and a significantly greater proportion of patients experiencing ≥50%, ≥70% and ≥90% reductions in monthly attack rate versus placebo, in patients receiving LTP with SC compared with IV C1INH (12).

Although the efficacy of C1INH SC has been proven through placebo-controlled clinical trials, there is a lack of real-world evidence of its effectiveness and use in a wide population of patients with HAE. Furthermore, besides small subgroup analyses and indirect treatment comparisons from trials, little is known as to whether C1INH SC has a benefit in terms of effectiveness over C1INH IV in the real world. The present study aimed to capture real-world evidence of the effectiveness of switching from other treatments to C1INH SC in patients with HAE-C1INH in Spain and Germany, by use of a retrospective single-cohort patient chart analysis.

## 2 Results

### 2.1 Patient characteristics

A total of 106 patient charts were included in the analysis: 69 from German centers, and 37 from Spanish centers. German patients were from Bavaria (16), Berlin (12), Hessen (8), Lower Saxony (2), Rhineland (13), North Rhine-Westphalia (10) and Schleswig Holstein (8); Spanish patients represented the regions Catalonia (3), Galicia (1), Madrid (23) and Andalucia (10). A summary of patient characteristics can be found in **Table 1**. One patient reported sex = ‘other’. For all analyses that utilized sex as a covariate, the patient was excluded to prevent the inclusion of an additional covariate and the accompanying reduction in degrees of freedom. In total, 48 patients (45.7%) had received IV C1INH LTP at baseline (68 [64.2%] received any LTP treatment) and 37 (35.2%) were treated on demand. In the overall population, mean treatment duration at baseline prior to switching was 5.6 months; patients received a mean of 12.6 months of treatment with C1INH SC during the post-switch period. Details of subgroups analyzed from the full sample are displayed in **Supplementary Table 1**.

**Table 1:** Baseline patient characteristics.

Characteristic	Sample statistic (N=106)
Country	
Germany	69
Spain	37
Age, mean years (SD)	34.57 (12.4)
Weight, mean kg (SD)	69.28 (13.7)
BMI, mean kg/m^2^ (SD)	24.74 (2.9)
Sex, n (%)	
Female	63 (60.0)
Male	42 (39.6)
Other*	1 (0.9)
Comorbidities, n (%)^†^	
Yes	44 (41.5)
No	62 (58.5)
Care at reference center, n (%)	
Yes	60 (56.6)
No	46 (43.4)
Baseline attack rate, n (%)	
<2 attacks/month	85 (80.2)
≥2 attacks/month	21 (19.8)
Baseline AAR, mean (SD)	13.8 (14.3)
Baseline treatment, n (%)	
pdC1INH IV LTP	44 (41.5)
pdC1INH IV LTP (Berinert^®^)^‡^ + Danazol	3 (2.8)
rhC1INH IV LTP^‡^	1 (0.9)
Danazol LTP	12 (11.3)
Stanozolol LTP	2 (1.9)
Tranexamic acid LTP	6 (5.7)
pdC1INH IV OD	12 (11.3)
rhC1INH IV OD	2 (1.9)
Icatibant + pdC1INH IV OD	2 (1.9)
Icatibant OD	21 (19.8)
Ecallantide^§^ OD	1 (0.9)
Baseline treatment duration, mean months (range)	5.6 (3.6)
C1INH SC treatment duration, mean months (range)	12.6 (6, 36.5)

*One patient reported sex = ‘other’. For all analyses utilizing sex as a covariate, the patient was excluded to prevent the addition of an additional covariate and the accompanying reduction in degrees of freedom. ^†^Comorbidities included depression, anxiety, cardiovascular disease and autoimmune abnormalities. ^‡^Not indicated for LTP in Europe; ^§^Ecallantide is not approved by the European Medicines Agency.

AAR, annualized attack rate; BMI, body mass index; EMA, European Medicines Agency; IV, intravenous; LTP, long-term prophylaxis; OD, on demand; pdC1-INH, plasma-derived C1 inhibitor; rhC1-INH, recombinant human C1 inhibitor; SC, subcutaneous; SD, standard deviation.

### 2.2 Reduced AAR after switching from IV C1INH LTP

**Figure 1:**
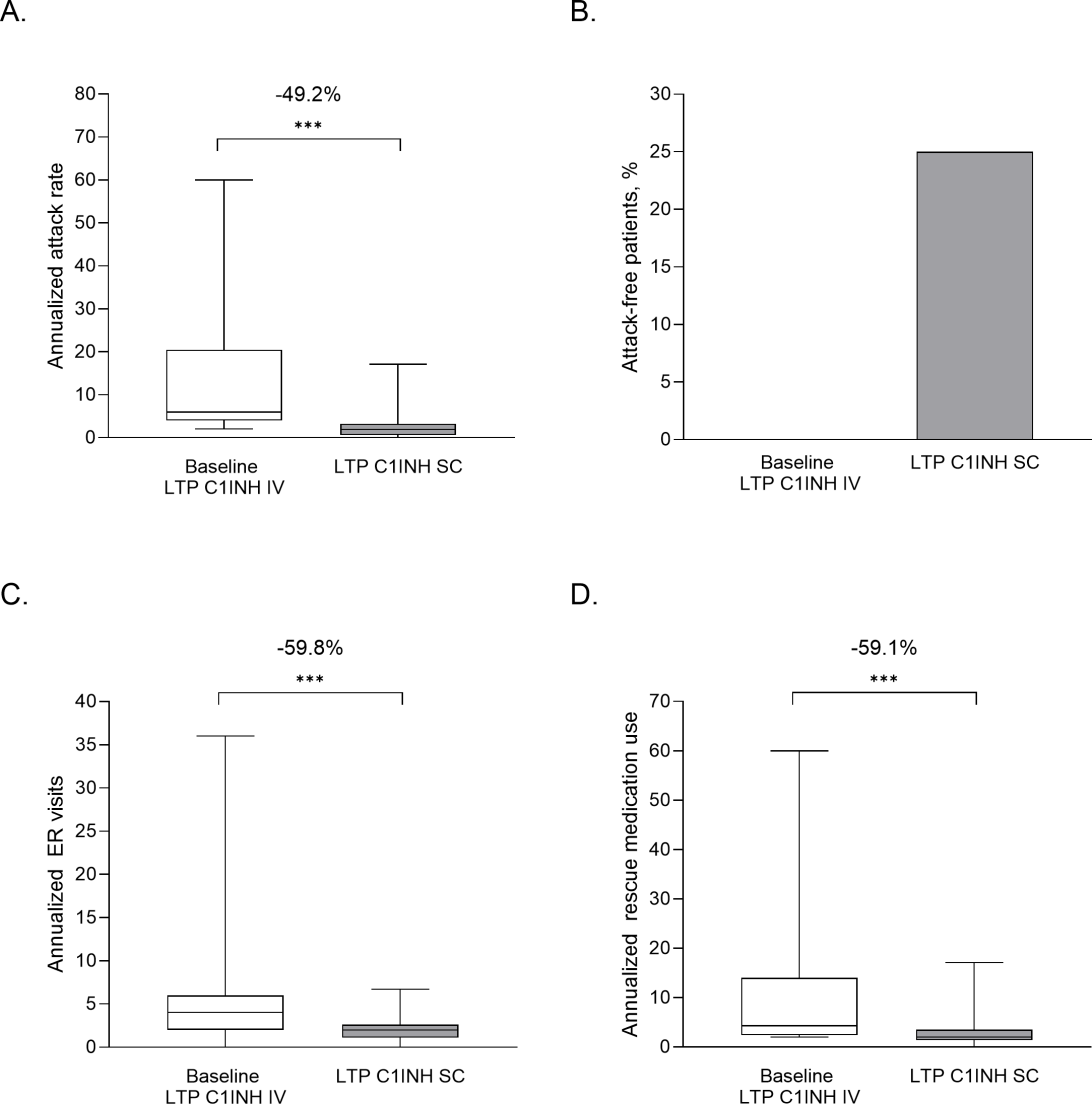
Clinical benefit of switching from IV to SC C1INH LTP in terms of AAR **(A)**, and attack-free patients **(B)**, ER visits **(C)** and rescue medication use **(D)**. Data are from patient charts (n=48) from the 6-month baseline period, where patients were treated with LTP C1INH IV, and ≥6 months after switching to LTP C1INH SC. Median, upper and lower quartile, and minimum and maximum values are represented. The mean percentage change as analyzed using LMM analysis are noted above; ***P<0.001. AAR, annualized attack rate; C1INH, C1 inhibitor; ER, emergency room; IV, intravenous; LTP, long-term prophylaxis; SC, subcutaneous.

In patients who switched from IV to SC C1INH LTP (n=48), the mean time-normalized number of HAE attacks (AAR; primary endpoint) was reduced by 12.3 from 15 attacks per year at baseline to 2.7 attacks per year during the C1INH SC treatment period (**Figure 1A**). Using a linear mixed model (LMM) regression analysis, this represented a change from baseline of -73.2% (95% confidence interval [CI] -1.57, -1.06; P<0.001; **Figure 1A**). The observed AAR reduction was similar using an alternate modeling process in which patients with an AAR of 0 in either period were not included in the analysis (73.0% reduction [n=36]; P<0.001). None of the patients receiving C1INH IV LTP at baseline were attack free, whereas 25% of patients were attack-free after switching to C1INH SC (P<0.001; **Figure 1B**). Data from Germany and Spain individually revealed statistically significant reductions in AAR following treatment switch in patients from IV to SC C1INH LTP from both countries. Baseline mean AAR reported for patients from Spain (35.5 attacks per year) were higher than those in Germany (8.1 attacks per year); however, both patients from Spain and Germany displayed a statistically significant reduction in AAR (P<0.001; **Supplementary Table 2**).

### 2.3 Reduced number of emergency room (ER) visits and rescue medication use after switching from IV C1INH LTP

Significant mean reductions were observed in the additional outcomes of ER visits and rescue medication use, in patients who switched from IV to SC C1INH LTP (n=48). The mean annualized ER or inpatient visits were reduced from 6.0 at baseline to 2.0 after switching – a 49.1% reduction (LMM analysis; **Figure 1C**; P<0.001). Mean annualized rescue medication use was reduced from 11.5 to 2.8 uses (59.1% by LMM; P<0.001; **Figure 1D**). When assessing individual country data, patients from Spain (n=12), as compared to patients from Germany (n=36), had more ER visits (10.0 vs 4.7/y) and more rescue medication use (30.3 vs 5.2/y) at baseline, but values following C1INH SC switch were reduced to similar levels in both countries (P<0.001 for both countries; **Supplementary Table 2**).

### 2.4 C1INH SC LTP was effective regardless of baseline treatment strategy and disease severity

**Figure 2:**
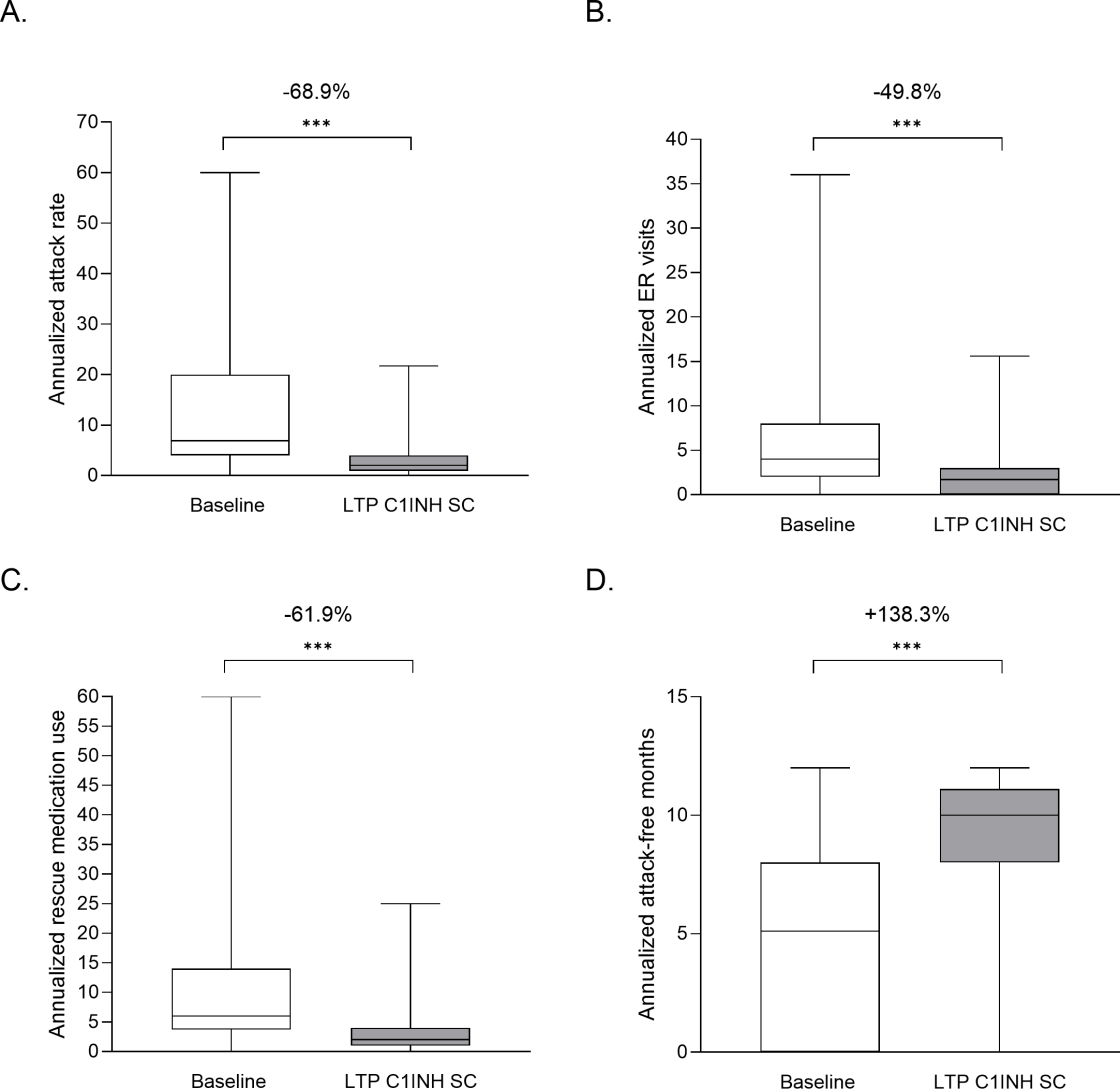
Outcome analyses of patients who switched from any treatment to LTP C1INH SC. Values are plotted for baseline and LTP C1INH SC treatment periods for annualized attack rate **(A)**, ER visits **(B)**, rescue medication use **(C)** and attack-free months **(D)**. Median, upper and lower quartile, and minimum and maximum values are represented. The mean percentage change as analyzed using LMM analysis are noted above; ***P<0.001. AAR, annualized attack rate; C1INH, C1 inhibitor; ER, emergency room; LTP, long-term prophylaxis; SC, subcutaneous.

In the full sample population (N=105), switching to C1INH SC LTP from any prior treatment was associated with significant mean reductions in AAR (13.8 vs 3.4 attacks per year; 68.9% reduction by LMM; P<0.001), ER visits (5.6 vs 2.0 per year; 49.8% reduction by LMM; P<0.001) and rescue medication use (12.1 vs 3.4 per year; 61.9% reduction by LMM; P<0.001) (**Figures 2A–C**). Due to the larger sample size, additional analyses on the full sample population were possible – namely, attack-free months and the proportion of patients who experienced <1 attack per month. The mean number of attack-free months increased following the switch to C1-INH SC LTP, from 4.5 months at baseline, to 8.9 months post-switch (138.3% increase by LMM; P<0.001; **Figure 2D**). Treatment with C1INH SC was also associated with a 31% increase in the proportion of patients within the sample with <1 attack per month, compared with baseline values (P<0.001; data not shown).

**Figure 3:**
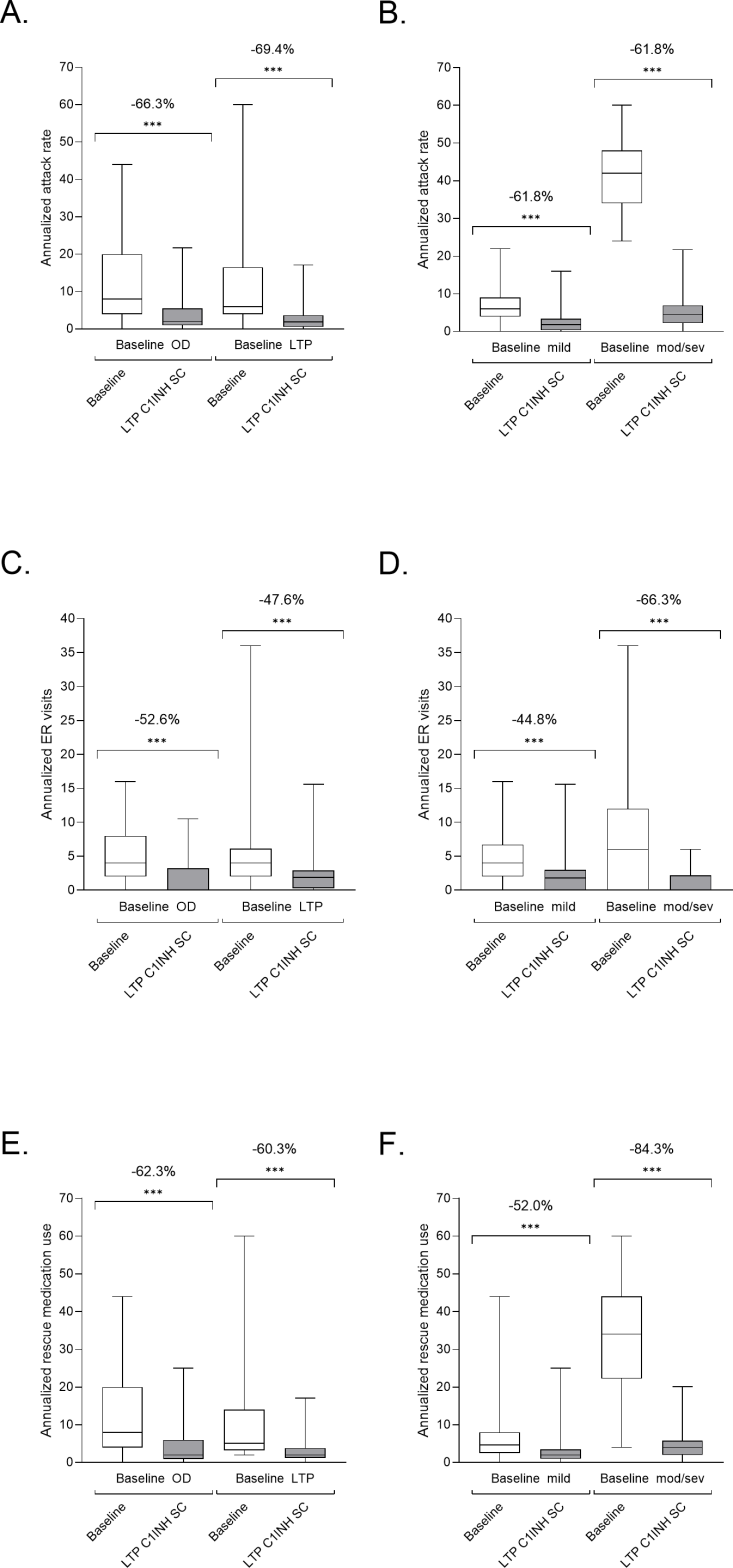
Subgroup analyses of patients with HAE that switched to LTP C1INH SC. Patients receiving OD or other LTP at baseline **(A, C, E)**, and those with <2 attacks per month or ≥2 attacks per month at baseline **(B, D, F)** were analyzed for annualized attack rate **(A, B)**, ER visits **(C, D)** and rescue medication use **(E, F)**. Median, upper and lower quartile, and minimum and maximum values are represented. The percentage change as analyzed using LMM analysis are noted above; ***P<0.001. Use of other LTP at baseline comprised pdC1INH IV, rhC1INH IV, danazol, stanozolol, and tranexamic acid. C1INH, C1 inhibitor; ER, emergency room; LTP, long-term prophylaxis; OD, on demand; SC, subcutaneous.

Subgroup analyses were performed on data from patients who switched from OD treatment and those who switched from an alternative LTP treatment, comprising pdC1INH IV, rhC1INH IV, danazol, stanozolol, and tranexamic acid. Similar improvement was observed, regardless of prior treatment strategy (**Figure 3**). Additionally, reductions in AAR, ER visits and rescue medication use were observed in both subgroups of patients with <2 attacks per month and ≥2 attacks per month at baseline. Patients with ≥2 attacks per month at baseline experienced a greater mean reduction in AAR (39.1 vs 6.4 per year; P<0.001 by LMM) than those with <2 attacks per month at baseline (7.4 vs 2.7 per year; P<0.001 by LMM). This was also the case for rescue use, where patients with ≥2 attacks per month had a higher mean baseline use of rescue medication (32.1) than those with <2 attacks per month (7.1); but post-switch to C1INH SC, both groups had similar rates of use (5.1 and 3.0, respectively) (**Figure 3**).

### 2.5 Observed associations between patient demographics and the efficacy of C1INH SC

Higher BMI values were associated with higher AARs (5.6% more per point; P=0.028), numbers of ER visits (5.0% per point; P=0.019) and use of rescue medication (7.2% per point; P=0.002) post-switch (**Table 2**). Frequency of ER visits was also higher in patients who had a comorbidity (55.0%; P<0.001; **Table 2**). Female sex was associated with higher AAR (Sex=male was associated with a 28.1% lower AAR, relative to female; P=0.032; **Table 2**). No outcome associations were observed with age (**Table 2**).

**Table 2:** Analysis of patient demographic associations with AAR, ER visits and rescue medication uses, using a log-level OLS model.

Outcome	Covariates	Coefficient	Confidence interval	% change	P-value
AAR	Age	0.01	(0.00, 0.02)	1.1	0.073
	Sex	-0.33	(-0.63, -0.03)	-28.1	**0.032**
	Comorbidity	0.13	(-0.16, 0.42)	13.5	0.397
	BMI	0.05	(0.01, 0.10)	5.6	**0.028**
ER visits	Age	-0.01	(-0.02, 0.00)	-0.6	0.232
	Sex	-0.05	(-0.32, 0.22)	-4.7	0.703
	Comorbidity	0.44	(0.17, 0.70)	55.0	**<0.001**
	BMI	0.05	(0.01, 0.09)	5.0	**0.019**
Rescue medication use	Age	0.01	(0.00, 0.02)	1.0	0.081
	Sex	-0.25	(-0.52, 0.02)	-22.3	0.066
	Comorbidity	0.19	(-0.07, 0.45)	20.7	0.160
	BMI	0.07	(0.03, 0.11)	7.2	**0.002**

Every year of age and every point on the BMI scale relates to the percentage change. Sex = male and comorbidity = yes relate to the percentage change. P<0.05 was considered significant; significant P-values are shown in bold.

AAR, annualized attack rate; BMI, body mass index; ER, emergency room; OLS, ordinary least squares.

### 2.6 Real-world dose and frequency of C1INH SC administration

Most patients were administered C1INH SC 8–9 times per month, with a median frequency of 8.0 per month (range 1.0–20.0). Median dose was 56.5 IU/kg (range 6.0–100 IU/kg) – close to the approved dose of 60 IU/kg (n=105; **Supplementary Table 3**). Patients from Germany received a median dose of 50 IU/kg (range 6.0–100.0) (n=69) and those in Spain received 60 IU/kg (range 17.8–60.0; n=37; **Supplementary Table 3**).

## 3 Discussion

This retrospective patient chart analysis found that patients with HAE who switched to C1INH SC experienced a significant reduction in AAR, ER/inpatient hospital visits and rescue medication use compared to the baseline period. Accordingly, switching to SC C1INH was also associated with increased attack-free months and an increased proportion of patients who experienced <1 attack per month, relative to baseline. It could be expected that those receiving OD treatment at baseline would benefit from switching to C1INH SC (or LTP generally); however, a significant clinical benefit was also observed in patients switching from other LTP therapies and, notably, even in those who were previously receiving C1INH IV for LTP. These data suggest that switching LTP therapies may help patients come closer to the ultimate goals of treatment – complete disease control and normalization of life (3).

This data supports the subgroup analysis of the COMPACT study, which showed that switching from C1INH IV LTP to 60 IU/kg SC C1INH LTP resulted in a clinically meaningful benefit in terms of HAE attack rate, with a reduction of 53.7% observed, although sample size was small (n=12) (11). A modeling study comparing the pharmacokinetics of IV (different formulation to the present study) and SC C1INH found that twice-weekly SC administration resulted in maintained functional levels of C1INH above the 40% threshold below which patients are more likely to experience attacks; whereas trough levels of functional C1INH reached 30% with IV administration (13), which could explain the reduction in attacks with SC versus IV C1INH LTP in the present study.

Based on mean attack-rate reductions of 51% in the CHANGE trial of C1INH IV LTP versus placebo (4, 12) and 84% overall in the COMPACT trial of 60 IU/kg C1INH SC LTP versus placebo (7, 12), an additional reduction of ~30% could have been expected from switching from C1INH IV LTP to SC LTP. However, in the current study, a greater AAR reduction of 73.2% was observed following the switch. The differences in patient populations between the clinical trial and the real world could be a reason for this. For example, the COMPACT trial excluded patients who received C1INH IV LTP in the run-in period (other HAE-specific LTPs were unavailable at the time), and both studies included only patients who experienced ≥2 attacks per month.

While the COMPACT trial reported a mean reduction in attack rate of 84% when patients previously receiving OD treatment only were treated with C1INH SC LTP (7), a reduction of 66.3% was observed in the current study. This may be in part explained by the abovementioned differences between trial and real-world patient populations, and patients recruited in the COMPACT trial may have had a greater baseline severity than the real-world population. In a trial setting, patients are guaranteed to receive treatment according to the protocol, whereas in real-world situations, patients may miss or delay doses, or be prescribed off-label doses according to attack frequency. From an author’s personal experience, in Spain it is common for some patients to bring forward their scheduled LTP dose as soon as they feel an attack is imminent, avoiding the use of rescue medication. It then becomes unclear as to whether this potential attack is reported or not. Since baseline attack frequency and rescue medication use varied greatly between the two countries, it appears that there may indeed be differences in attack perception, and whether all attacks are treated with rescue medication, or only more severe/debilitating attacks, as was historically recommended (14). Some patients in Spain may also delay doses as the attack-free interval increases due to increased confidence, until a breakthrough attack acts as a reminder to adhere to the initial dosing protocol (author experience). Furthermore, physicians in Spain follow the recommendations of the Spanish Bradykinin Angioedema Group (GEAB), which recommend that patients should start with a dose of 2000 IU twice weekly (15). If the patient experiences a partial response, the dose is increased to 2000 IU three times a week. In severe cases, it is recommended to start with the label dose of 60 IU/kg twice weekly. This could mean that some patients do not reach the recommended dose. Several associations were observed between outcomes and patient characteristics. Higher BMI was associated with increased AAR, ER visits and rescue medication use in the current study. BMI has not previously been associated with increased attack frequency but has been linked to attack severity (16). Studies have demonstrated that there is a negative relationship between body weight and steady-state C1INH trough levels following treatment with C1INH SC (17). Patients with a low body weight are predicted to achieve a higher functional C1INH level compared with patients with a higher body weight administered with a fixed dose of C1INH (17). Therefore, body weight-adjusted dosing, currently only possible for LTP with C1INH SC, aims to achieve the same level of activity across a range of body weights.

A new treatment paradigm is emerging where patients and physicians are encouraged to aim for zero attacks and to live a normal life (3, 18). Recently, an expert panel agreed by Delphi consensus on several factors that should be considered when assessing whether HAE is well controlled, including time-normalized number of attacks, the requirement for rescue medication, the proportional reduction in the number of attacks with treatment and the number of emergency department visits and hospitalizations (18). The panel also agreed that the mean length of attack-free periods, hours of activity impairment and the number of days of sick leave were factors that should be considered when assessing whether a patient’s life is normal (18). As such, these data are supportive for the use of C1INH SC LTP in all patients regardless of HAE severity or those previously receiving alternative LTP therapies as substantial reductions in AAR, ER visits and rescue medication use were observed following the switch to C1INH SC LTP. Switching LTP from IV to SC C1INH may help to bring all patients closer to achieving the ultimate goals of HAE treatment (18).

The current WAO/EAACI guideline for the management of HAE recommends that all patients are considered for LTP at all visits, and that patients are monitored frequently (3). An individualized action plan should be developed by shared decision making between the patient and physician (3). Patients who are more engaged in shared decision making have better treatment adherence (19) and are more likely to proactively request a treatment switch (20). Although patient preference should be considered, accessibility and cost-effectiveness of available specific LTP treatments play a major role in treatment decision making in centers (author experience). OD treatment options are accompanied by large direct costs associated with treatment, hospitalizations and treatment of related psychological comorbidities, predicted to be 363,795 USD per patient per year, with predicted socio-economic costs of 52,576 USD per patient per year (21). This real-world data shows that treatment with C1INH SC LTP can reduce the need for OD treatment and reduce hospitalizations by around 50%. Clinical trial data have shown clinically meaningful improvements in QoL scores following treatment with C1INH SC LTP, including on the Hospital Anxiety and Depression (HADS) scale (9, 10). Further studies are required to determine QoL benefits, patient satisfaction and cost-effectiveness regarding C1INH SC LTP use in the real world.

## 4 Data limitations and perspectives

A potential limitation of the study is sample bias, as the mechanism of collecting patient charts from providers may not capture the true product use for each patient. For example, patients may not report all OD use, and missed doses may not be consistently reported. Although patients should follow their prescription and provider’s instructions for C1INH SC use, patients may forget, skip, bring forward or delay treatment in real-world situations. Interviews, checking patient charts for erroneous entries and callbacks for missing data were performed, which aimed to reduce bias. Another limitation is that baseline IV C1INH LTP dose was not recorded in the study; since there are differences in posology between products, fixed dosing could affect efficacy versus weight-based dosing. Due to differences in treatment access, market power, compliance oversight and healthcare expenditures between reference and non-reference centers, the average dose per patient of C1INH SC may differ between the two types of center. A reference center covariate was included in analysis to minimize sample bias due to potential treatment differences.

## 5 Conclusions

In the absence of head-to-head studies, this real-world data from Germany and Spain provides the first evidence that patients may derive substantial reductions in attack rate, hospitalizations and rescue medication use by switching from IV to SC C1INH for LTP. Patients from Germany and Spain experienced reductions in these three key criteria in assessing disease control and normalization of patients’ lives, regardless of prior treatment and disease severity.

## 6 Materials and Methods

### 6.1 Study design

A retrospective, multicenter, non-interventional analysis was conducted using patient charts obtained from HAE references centers in Germany and Spain. Allergists from participating centers provided patient charts covering the period February 2019 to February 2022 (36 months). Data were collected regarding annualized attack rate (AAR), frequency of administration, dose volume, patient demographics (age, weight, sex and comorbidity status), referring physician type, length of treatment, prior medications, current rescue medications, prior rescue medications, and covariates. Data were obtained from a baseline period, defined as the six months prior to switching to C1INH SC LTP, and during the treatment period of ≥6 consecutive months. At baseline, patients who had been on LTP therapy for ≥3 months were considered an ‘LTP’ patient for subsegment analyses.

### 6.2 Inclusion and exclusion criteria

Patients were included if they were clinically diagnosed with HAE-C1INH type 1 or 2 and had received C1INH SC (Berinert^®^ 2000/3000) for LTP for at least six continuous months during the period between February 2019 and February 2022. Patients were excluded if they were enrolled in any clinical trial for HAE during the three months prior to starting treatment with or during treatment with C1INH SC LTP.

### 6.3 Outcome measures

The primary endpoint was the patients’ change in AAR between the 6-month baseline period in which patients utilized LTP IV C1INH compared with the subsequent period in which the patients used C1INH SC LTP. AAR was defined as the annualized number of physician-reported attacks as documented on the patient chart. Secondary endpoints were the mean AAR, ER visits and rescue medication uses for the whole sample population, and subpopulations based on baseline disease severity and baseline treatment. Annual ER visits or inpatient hospitalizations were defined as those due to HAE; a visit does not imply that an attack occurred or that rescue medication was used. Rescue medication use was defined as use of OD medication regardless of where the patient is on an LTP regimen, reported as an annualized value.

Minimum attack-free months was assessed as the minimum number of full months in a year that the patient could have been attack free during the baseline or treatment period with C1INH SC LTP (higher value = longer attack-free period). The proportion of patients experiencing <1 attack per month was also assessed. Associations between patient demographics and AAR were analyzed, in addition to between-subject analyses, in order to assess differences in C1INH SC LTP efficiency and utilization across patient demographics.

### 6.4 Statistical analysis

Statistical analyses were performed using Stata^®^ version 17.0. Comparisons between baseline and post-switch AAR, ER visits, rescue medication use, and minimum attack-free months were assessed using log-level LMM regression analyses, in which the outcome variable (change from baseline) was log transformed by ln(Y+1), with fixed effects used to control for patient demographics (age, sex, body mass index [BMI], comorbidity status), and to control for treatment parameters (country). Log-level LMMs with AAR = 0 values removed provided a robustness check to ensure the translation was not a source of bias. A level-level LMM was used to analyze the proportion of patients with <1 attack per month. A proportions test was used to assess attack-free patients. Mean of differences were calculated in a paired t-test. A log-level ordinary least squares model was used to analyze the effect of patient demographic on outcomes and utilization. P<0.05 was considered a significant association. Included fixed effects and definitions included: age – as of February 1, 2019; sex – the biological sex of the patient at birth; comorbidity status – a binary variable describing if the patient had any of the study-defined comorbidities (depression, anxiety, cardiovascular disease, and autoimmune disorders); BMI – the BMI of the patient, measured both during the baseline and treatment period; Spain – a binary variable describing if the patient received treatment in Spain, with the alternative being Germany.

## Data Availability

The raw data supporting the conclusions of this article will be made available by the authors, without undue reservation.

## References

[1] Magerl M Sala-Cunill A Weber-Chrysochoou C Trainotti S Mormile I Spadaro G. Could it be hereditary angioedema?-Perspectives from different medical specialties. Clin Transl Allergy. (2023) 13:e12297. doi: 10.1002/clt2.12297 37746796 PMC10509412

[2] Farkas H. Hereditary angioedema: examining the landscape of therapies and preclinical therapeutic targets. Expert Opin Ther Targets. (2019) 23:457–9. doi: 10.1080/14728222.2019.1608949 31018718

[3] Maurer M Magerl M Betschel S Aberer W Ansotegui IJ Aygoren-Pursun E. The international WAO/EAACI guideline for the management of hereditary angioedema-The 2021 revision and update. Allergy. (2022) 77:1961–90. doi: 10.1111/all.15214 35006617

[4] Zuraw BL Busse PJ White M Jacobs J Lumry W Baker J. Nanofiltered C1 inhibitor concentrate for treatment of hereditary angioedema. N Engl J Med. (2010) 363:513–22. doi: 10.1056/NEJMoa0805538 20818886

[5] Kalaria S Craig T. Assessment of hereditary angioedema treatment risks. Allergy Asthma Proc. (2013) 34:519–22. doi: 10.2500/aap.2013.34.3702 24169059

[6] Riedl MA Banerji A Busse PJ Johnston DT Davis-Lorton MA Patel S. Patient satisfaction and experience with intravenously administered C1-inhibitor concentrates in the United States. Ann Allergy Asthma Immunol. (2017) 119:59–64. doi: 10.1016/j.anai.2017.05.017 28668241

[7] Longhurst H Cicardi M Craig T Bork K Grattan C Baker J. Prevention of hereditary angioedema attacks with a subcutaneous C1 inhibitor. N Engl J Med. (2017) 376:1131–40. doi: 10.1056/NEJMoa1613627 28328347

[8] Behring CSL. Berinert^®^ 2000/3000 Summary of Product Characteristics. (2021). Available online at: https://www.medicines.org.uk/emc/product/13819/smpc.

[9] Lumry WR Zuraw B Cicardi M Craig T Anderson J Banerji A. Long-term health-related quality of life in patients treated with subcutaneous C1-inhibitor replacement therapy for the prevention of hereditary angioedema attacks: findings from the COMPACT open-label extension study. Orphanet J Rare Dis. (2021) 16:86. doi: 10.1186/s13023-020-01658-4 33588897 PMC7885603

[10] Lumry WR Craig T Zuraw B Longhurst H Baker J Li HH. Health-Related quality of life with subcutaneous C1-inhibitor for prevention of attacks of hereditary angioedema. J Allergy Clin Immunol Pract. (2018) 6:1733–41 e3. doi: 10.1016/j.jaip.2017.12.039 29391286

[11] Craig T Lumry W Cicardi M Zuraw B Bernstein JA Anderson J. Treatment effect of switching from intravenous to subcutaneous C1-inhibitor for prevention of hereditary angioedema attacks: COMPACT subgroup findings. J Allergy Clin Immunol: In Pract. (2019) 7:2035–8. doi: 10.1016/j.jaip.2019.01.007 30660873

[12] Bernstein JA Li HH Craig TJ Manning ME Lawo JP Machnig T. Indirect comparison of intravenous vs. subcutaneous C1-inhibitor placebo-controlled trials for routine prevention of hereditary angioedema attacks. Allergy Asthma Clin Immunol. (2019) 15:13. doi: 10.1186/s13223-019-0328-3 30899278 PMC6407188

[13] Pawaskar D Tortorici MA Zuraw B Craig T Cicardi M Longhurst H. Population pharmacokinetics of subcutaneous C1-inhibitor for prevention of attacks in patients with hereditary angioedema. Clin Exp Allergy. (2018) 48:1325–32. doi: 10.1111/cea.2018.48.issue-10 29998524

[14] Craig T Pürsün EA Bork K Bowen T Boysen H Farkas H. WAO guideline for the management of hereditary angioedema. World Allergy Organ J. (2012) 5:182–99. doi: 10.1097/WOX.0b013e318279affa PMC3651186 23282420

[15] Grupo Español de Estudio del Angioedema mediado por Bradicinina (GEAB) de la Sociedad Española de Alergología e Inmunología Clínica (SEAIC). Protocolo de Incio de Tratamiento con BERINERT^®^ Subcutaneo como Profilaxis a Largo Plazo en Pacientes con Angioedema Hereditario por Deficit de C1-Inhibitor (AEH-C1-INH). (2020). Available online at: https://www.seaic.org/profesionales/noticias-para-profesionales/protocolo-inicio-berinert-subcutaneo.html.

[16] Caballero T Zanichelli A Aberer W Maurer M Longhurst HJ Bouillet L. Effectiveness of icatibant for treatment of hereditary angioedema attacks is not affected by body weight: findings from the Icatibant Outcome Survey, a cohort observational study. Clin Transl Allergy. (2018) 8:11. doi: 10.1186/s13601-018-0195-x 29599966 PMC5870812

[17] Zuraw BL Cicardi M Longhurst HJ Bernstein JA Li HH Magerl M. Phase II study results of a replacement therapy for hereditary angioedema with subcutaneous C1-inhibitor concentrate. Allergy. (2015) 70:1319–28. doi: 10.1111/all.2015.70.issue-10 PMC4755045 26016741

[18] Maurer M Aygoren-Pursun E Banerji A Bernstein JA Balle Boysen H Busse PJ. Consensus on treatment goals in hereditary angioedema: A global Delphi initiative. J Allergy Clin Immunol. (2021) 148:1526–32. doi: 10.1016/j.jaci.2021.05.016 34048855

[19] Babac A von Friedrichs V Litzkendorf S Zeidler J Damm K Graf von der Schulenburg JM. Integrating patient perspectives in medical decision-making: a qualitative interview study examining potentials within the rare disease information exchange process in practice. BMC Med Inf Decis Making. (2019) 19:188. doi: 10.1186/s12911-019-0911-z PMC6751820 31533712

[20] Riedl MA Neville D Cloud B Desai B Bernstein JA. Shared decision-making in the management of hereditary angioedema: An analysis of patient and physician perspectives. Allergy Asthma Proc. (2022) 43:397–405. doi: 10.2500/aap.2022.43.220050 35820771

[21] Castaldo AJ Jervelund C Corcoran D Boysen HB Christiansen SC Zuraw BL. Assessing the cost and quality-of-life impact of on-demand-only medications for adults with hereditary angioedema. Allergy Asthma Proc. (2021) 42:108–17. doi: 10.2500/aap.2021.42.200127 PMC8133018 33581742

