# Supplementary material for: Real-world evidence of the effectiveness and utilization of subcutaneous C1INH long-term prophylaxis in patients with HAE in Spain and Germany
Source: Front Immunol. 2025 May 14;16:1576235. doi: 10.3389/fimmu.2025.1576235 (PMC12116583; doi:10.3389/fimmu.2025.1576235)
Supplement: Supplementary file 1 [file DataSheet1.docx]

## Supplementary Data

**Supplementary Table 1.** Subgroups for analysis.

| **Subgroup** | **N** | **Mean (SD) baseline AAR (n/year)** | **Mean (SD) dose of  C1INH SC LTP (IU/kg)** | **Mean (SD) dosing frequency of C1INH SC LTP (doses/month)** |  |
| --- | --- | --- | --- | --- | --- |
| **Full sample^*^** | **105** | **13.8 (14.3)** | **45.9 (20.9)** | **6.8 (3.4)** |  |
| Baseline IV C1INH LTP | 48 | 15.0 (16.6) | 48.2 (20.1) | 7.7 (3.6) |  |
| Baseline OD regimen | 37 | 14.0 (12.4) | 45.1 (22.3) | 5.5 (3.0) |  |
| Baseline LTP regimen | 68 | 13.7 (15.3) | 46.3 (20.2) | 7.5 (3.4) |  |
| Baseline <2 attacks/month | 84 | 7.4 (5.5) | 47.5 (21.7) | 6.3 (3.3) | |
| Baseline ≥2 attacks per month | 21 | 39.1 (9.7) | 39.4 (16.3) | 8.8 (3.2) |  |

^*^Excludes one patient with ‘other’ sex to ensure maximal degrees of freedom in analyses.
AAR, annualized attack rate; C1INH, C1 inhibitor; IU, international units; IV, intravenous; LTP, long-term prophylaxis; OD, on demand; SC, subcutaneous.

**Supplementary Table 2.** AAR, ER and rescue medication use in patients that switched from C1INH IV LTP to C1INH SC LTP: Subgroup analysis of patients from Germany and Spain.

|  |  | **AAR (n/y)** | **ER visits (n/y)** | **Rescue medication use (n/y)** |
| --- | --- | --- | --- | --- |
| Mean baseline C1INH IV LTP | Germany | 8.1 | 4.7 | 5.2 |
|  | Spain | 35.5 | 10.0 | 30.3 |
| Mean C1INH SC LTP | Germany | 2.0 | 2.2 | 2.2 |
|  | Spain | 4.8 | 1.2 | 4.5 |
| Mean reduction | Germany | 6.1 | 2.5 | 3.0 |
|  | Spain | 30.7 | 8.8 | 25.8 |
| Mean % reduction | Germany | 75.8 | 52.6 | 57.9 |
|  | Spain | 86.3 | 87.6 | 85.1 |
| P value* | Germany | <0.001 | <0.001 | <0.001 |
|  | Spain | <0.001 | <0.001 | <0.001 |

*P value calculated through a mean of differences in a paired t-test.
AAR, annualized attack rate; C1INH, C1 inhibitor; ER, Emergency Room; IV, intravenous; LTP, long-term prophylaxis; SC, subcutaneous.

**Supplementary Table 3.** Full sample data for the effectiveness and utilization of C1INH SC in all patients (N=105), patients in Germany (n=69) and patients from Spain (n=37).

| **Statistics** |  | **Median  (range)** | **Mean  (SD)** |
| --- | --- | --- | --- |
| AAR (n/y) | All patients | 2.0 (0.0–21.7) | 3.9 (4.4) |
|  | Germany | 1.8 (0.0–21.7) | 2.7 (3.4) |
|  | Spain | 2.2 (0.0–19.0) | 4.8 (5.5) |
| ER visits (n/y) | All patients | 1.6 (0.0–15.6) | 2.0 (2.6) |
|  | Germany | 2.0 (0.0–10.5) | 2.2 (2.0) |
|  | Spain | 0.0 (0.0–15.6) | 1.6 (3.4) |
| Rescue medication  uses (n/y) | All patients | 2.0 (0.0–25.0) | 3.4 (4.2) |
|  | Germany | 2.0 (0.0–25.0) | 3.3 (4.0) |
|  | Spain | 2.0 (0.0–17.1) | 3.7 (4.5) |
| Attack-free months | All patients | 10.0 (0.0–12.0) | 8.9 (3.4) |
|  | Germany | 10.2 (0.0–12.0) | 9.5 (2.7) |
|  | Spain | 9.8 (0.0–12.0) | 7.8 (4.3) |
| Dose (IU/kg) | All patients | 56.5 (6.0–100.0) | 45.9 (20.9) |
|  | Germany | 50.0 (6.0–100.0) | 45.2 (23.4) |
|  | Spain | 60.0 (17.8–60.0) | 47.2 (15.4) |
| Frequency (doses/month) | All patients | 8.0 (1.0–20.0) | 6.8 (3.4) |
|  | Germany | 6.0 (1.0–20.0) | 6.0 (3.9) |
|  | Spain | 8.0 (3.0–12.0) | 8.2 (1.6) |

AAR, annualized attack rate; C1INH, C1 inhibitor; ER, Emergency Room; IU, international units; n/y, number per year.
